# The Tennessee Department of Health WORKshops on Use of Secondary Data for Community Health Assessment, 2012

**DOI:** 10.5888/pcd11.130206

**Published:** 2014-01-02

**Authors:** Bruce A. Behringer, Ellen Omohundro, Derrick Boswell, Dwayne Evans, Lori B. Ferranti

**Affiliations:** Author Affiliations: Ellen Omohundro, Derrick Boswell, Dwayne Evans, Lori B. Ferranti, Tennessee Department of Health, Nashville, Tennessee.

## Abstract

Community health assessment is a core function of public health departments, a standard for accreditation of public health departments, and a core competency for public health professionals. The Tennessee Department of Health developed a statewide initiative to improve the processes for engaging county health departments in assessing their community’s health status through the collection and analysis of secondary data. One aim of the Tennessee Department of Health was to position county public health departments as trusted leaders in providing population data and engaging community stakeholders in assessments. The Tennessee Department of Health’s Division of Policy, Planning, and Assessment conducted regional 2-day training workshops to explain and guide completion of computer spreadsheets on 12 health topics. Participants from 93 counties extracted data from multiple and diverse sources to quantify county demographics, health status, and resources and wrote problem statements based on the data examined. The workshops included additional staff development through integration of short lessons on data analysis, epidemiology, and social-behavior theory. Participants reported in post-workshop surveys higher degrees of comfort in interpreting data and writing about their findings on county health issues, and they shared their findings with health, hospital, school, and government leaders (including county health council members) in their counties. Completion of the assessments enabled counties and the Tennessee Department of Health to address performance-improvement goals and assist counties in preparing to meet public health accreditation prerequisites. The methods developed for using secondary data for community health assessment are Tennessee’s first-phase response to counties’ request for a statewide structure for conducting such assessments.

## Introduction

Community health assessment (CHA) is a long-standing core public health function (1) and is now a prerequisite activity for public health accreditation (2) and a core competency for public health professionals (3). Through the Patient Protection and Affordable Care Act of 2010, CHA became a required activity for nonprofit, tax-exempt hospitals (4). CHA processes aim to identify data on population health used to determine needs and priorities for community health-improvement plans. The repository for much of population health data is the state health department. Making data accessible and understandable to local public health staff and translatable to community stakeholders is a challenge in CHA.

The Tennessee Department of Health (TDH) recognizes the necessity of understanding community health needs in a way that supports data-driven decision making at the county, regional, and state levels. Realizing the challenging environment of health care reform and resulting new demands on public health professionals, county and regional health department representatives requested in November 2011 that TDH leadership re-engage a statewide CHA. County health departments desired to reassert a leadership role in population-based assessment and use CHA results to develop action plans with local stakeholders. Because the most recent county-specific statewide assessment took place in the mid-1990s, county and state public health officials viewed this re-engagement as important to empower local leaders to identify and respond to today’s public health needs.

The request for a statewide CHA dovetailed with a new departmental priority to promote organizational continuous improvement. TDH introduced the Baldrige Performance Excellence approach (5) in November 2011 to assist the department in viewing all parts of the state, regional, and county health departments as “internal customers.” The counties’ request for a standardized, statewide CHA process became a means to address 3 of the 7 Baldrige criteria: strategic planning, customer service and measurement, and analysis and knowledge management (Figure). These 7 interrelated criteria form a framework of organizational processes that promote a comprehensive view for planning the improvement of organizational performance. The CHA demonstrates cooperative engagement between state and county health department staffs and is an important preparatory step for public health accreditation by the Public Health Accreditation Board.

**Figure:**
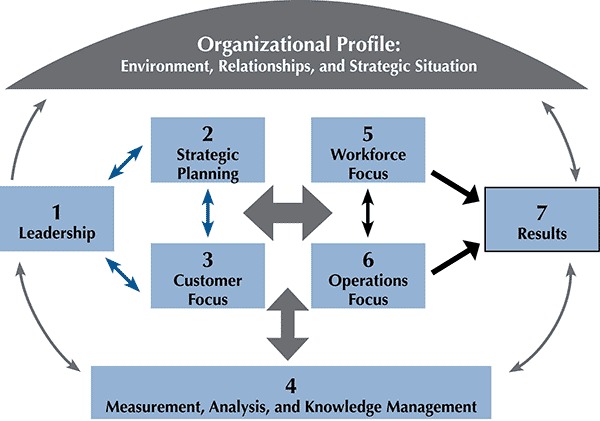
Baldrige criteria (6).

As an award recipient of the National Public Health Improvement Initiative (NPHII) Strengthening Public Health Infrastructure for Improved Health Outcomes (7) grant program, TDH had a mechanism to support such a statewide effort. The NPHII grant promotes and encourages goals similar to the goals of the Tennessee CHA: 1) improve capacity within health departments for evaluating effectiveness of organizations, practices, partnerships, programs, and use of resources through performance management; 2) expand and train public health staff and community leaders to conduct policy activities in key areas, and facilitate improvements in system efficiency; and 3) maximize the public health system to improve networking, coordination and cross-jurisdictional cooperation for the delivery of public health services to address resource sharing and improve health indicators (7).

The primary goal of the CHA was to improve the processes, findings, and use of CHAs as a competency of county health departments in Tennessee. Objectives were —

1. To develop a CHA tool that uses existing secondary data to produce consistent health information.
2. To improve state assistance to counties by making population health data more accessible and understandable.
3. To promote community benefits through shared findings with stakeholders to identify priorities for health improvement plans.
4. To engage participants in a replicable, experiential learning approach, including collegial exchange, to improve statewide public health competency in assessment.
5. To create an environment in which use of data is expected for planning, grant proposal writing, and program evaluation.

## Designing the Elements of the Community Health Assessment

To meet its objectives, TDH needed to design a CHA program that a range of county health department professionals (including field-based nurses, health educators, county directors, and epidemiologists) could complete quickly and easily. The program needed to consist of a single, standardized method, and it needed to be replicable to encourage future use. Lastly, the program needed to create an environment that encouraged participants to identify collaboratively common challenges, available resources, and opportunities for improvements. 

### Planning and module preparation

First, staff from the Division of Policy, Planning and Assessment (PPA) formed a workgroup with members from 4 county health departments with previous experience in conducting a CHA (Box 1). The workgroup 1) affirmed the desire to use a single standardized method across the state, 2) proposed a computer-assisted workshop approach to engage learners in finding and analyzing their own county-specific data while learning about data and their uses, and 3) recommended an environment that encouraged comparisons of data across geographic areas. Training sessions were called “WORKshops” to indicate an expectation of active learning and completion of an assessment as a product of participants’ time and effort. Initially, the workgroup selected 25 potential topics for the CHA. Results from 112 brief surveys among the state’s 95 counties helped prioritize a list of 17 health topics; a final set of 12 topics was selected, and a sample workshop module was designed for the topic *cancer*. Workgroup members also submitted suggestions for useful data and websites that they had used in conducting their own assessment.

**Box 1. Departmental Human Resources Tapped for Community Health Assessment**

- Planning workgroup members from 4 county health departments
- Epidemiology and statistical staff from the Division of Policy, Planning and Assessment to access data and use technology to develop modules
- Content experts from the Tennessee Department of Health programs to advise on module content
- Deputy Commissioner for Continuous Improvement and Training as faculty
- Participants from 93 county health departments

Next, TDH’s leadership team and regional and metropolitan county health department directors reviewed the proposal for conducting a statewide CHA using secondary data. Leadership identified important aspects of the proposed assessment. The assessment would enable counties to collect, analyze, and present data and increase their awareness of differences and disparities between county, state, and national data indicators. The assessment would also empower county staffs to share findings with their county health councils (established in 1996 [8]) and to help councils identify local health priorities. Participation in the CHA among Tennessee’s 95 county health departments would be voluntary. As part of the proposal, the workshop designers demonstrated the sample module on cancer. This module required participants to access county and state data for cancer rates (from the National Cancer Institute’s State Health Profiles) and risk factors (from the Behavioral Risk Factor Surveillance System [BRFSS]). The module also required participants to fill in a short inventory of cancer-related prevention and treatment resources and services available in the county and to answer “trigger questions,” which were designed to elicit reflective and comparative analysis using extracted data and local county knowledge. After the sample format was approved, PPA staff used it to create modules for the remaining 11 topics.

Working with 27 content experts from TDH, PPA staff led the process of module formulation and technical development beginning in April 2012. Content experts contributed topic-specific materials, including 1) sample learning objectives; 2) key topical issues of which each county should be aware; 3) relevant data indicators and websites; 4) types of locally available information on county resources and situations; 5) trigger questions about county data and comparisons with state and national data; and 6) supplemental information.

PPA staff used this input to create 12 modules formatted in Microsoft Excel (Microsoft Corp, Redmond, Washington) (Box 2). Staff identified 557 data points (eg, disease rates, demographic percentages, BRFSS measures) from 18 data sources and 14 websites. Additional non-Web–based data sources were provided by TDH business units and data systems. Staff checked each website and data source to confirm their availability, updated hyperlinks, and developed and checked Excel cell formulas to ensure that workshop participants would be able to complete their tasks accurately. Trigger questions in each module required participants to write statements comparing their county data with state and national data. Planners designed a 2-day agenda after developing and testing the modules.

**Box 2. Technical Components of Module Development**

- Modules developed in Microsoft Excel (Microsoft Corp, Redmond, Washington)
- Excel VBA (Visual Basic for Applications) objects and formulas used to create interactive, multipage, interlinked applications
- Hyperlinks to Web addresses and embedded Tennessee Department of Health reports led to data
- Supplemental topical files provided in PDF attachments
- Non-Web–based data sources developed from Tennessee Department of Health business units and data systems

### Workshop implementation

Three CHA workshops were conducted in June 2012, 1 each in the eastern, middle, and western regions of the state. This geographic distribution facilitated attendance, enabled interaction among peers from the same region, and reduced travel expenses. PPA staff coordinated logistics, including identifying locations with computer labs, preparing meeting materials, and arranging hotel accommodations. TDH Division of Local Health Services helped to recruit counties to attend the workshops. The deputy commissioner made presentations at regional county health councils meetings and promoted the CHA at Tennessee Public Health Association regional meetings.

The first day of the workshop opened with a videotaped welcome by the commissioner of health, who indicated the importance of the CHA as part of organizational performance improvement. On the first day, participants completed the following modules: 1) demographics (introducing data used in subsequent modules); 2) local health department services; 3) risk factors (largely based on BRFSS data); 4) cancer; 5) diabetes; 6) heart disease and stroke; and 7) health disparities. The second day began with a summary and debriefing of modules covered during the first day. The state director of vital statistics presented “The Art of Data” to explain how to interpret data and ways to present data to support data-driven decision making. Next, participants completed the remaining modules: 8) access to care; 9) oral health and dental care; 10) mental health; 11) perinatal issues and infant mortality; and 12) child health. At the conclusion of the final module on the second day, participants wrote problem statements using data from the completed modules.

A moderator introduced each module by using PowerPoint slides and describing the topic’s importance to the health of the state’s population. The deputy commissioner and PPA staff integrated basic epidemiologic concepts and health behavior theories as part of module introductions. Participants committed to share these findings with supervisors and county health councils on return to their counties. Participants left the workshop with paper copies of all modules and a USB flash drive containing their completed modules, supplemental topical information (readings and Web references), and supporting secondary data sets. PPA staff informed participants that they would follow up with them for evaluation purposes and that a CHA, consisting of more in-depth assessment methods, would start in fall 2012.

## Workshop Participation, Outcomes, and Evaluation

In all, 93 of 95 counties participated in the workshops; participating counties completed 98% of all modules (Table 1) and wrote 388 problem statements. Participants completed a short pre-test immediately before beginning the first day of the workshop; the same questions were used in a post-test at the end of the second day of the workshop. Nine months later, an evaluation was conducted via e-mail of participant satisfaction with the workshop and use of assessment data.

**Table 1:** Percentage of Counties (n = 93) That Completed Modules During Workshops on Using Secondary Data for Community Health Assessments, Tennessee, 2012

Module	Percentage of Counties That Completed Module
Demographics of county	100
County health department services	97
Risk factors	100
Cancer	95
Diabetes	97
Heart disease and stroke	96
Health disparities	98
Access to care	100
Oral health and dental care	98
Mental health	100
Perinatal issues and infant mortality	91
Child health	100

The pre-test identified frustration among participants with using secondary data. Participant concerns included limited access to timely county-specific data, the labor-intensive process of locating and extracting data, and difficulties in interpreting data. Participants also reported lack of awareness of many websites and TDH data resources. The post-test indicated that the most important workshop outcomes were that participants had learned where to find data, who to call at TDH, and how to interpret data. One participant stated, “We should look at the data, not just address the numbers that are being requested.” Pre-test to post-test comparisons indicated that comfort levels in working with data increased significantly by the end of the workshop (Table 2).

**Table 2:** Change in Comfort Levels of Working With Data After Workshops on Using Secondary Data for Community Health Assessments, Tennessee, 2012^a^

On a scale of 1 to 5, how comfortable are you now in . . .	Often Use This Skill, %	Pre-Test Mean^b^	Post-Test Mean^b^	*P* Value^c^
Finding data on the Internet	61.8	3.7	4.2	<.001
Calculating and comparing numbers, percentages, and rates	40.2	3.2	4.0	<.001
Creating charts, graphs, and maps with data	17.6	2.8	3.6	<.001
Writing problem statements using data	26.4	2.9	3.6	<.001
Making presentations/teaching using data	44.6	3.6	4.0	<.001
Making PowerPoint data presentations	39.8	3.4	4.0	<.001

a Survey results are presented only for participants who reported often using the skill.

b On a scale of 1 to 5, with 5 being most comfortable.

c Based on paired-samples *t* tests.

The pre- and post-test asked participants to state 3 ways their county’s health-related data differed from those for other counties and from those for the United States as a whole. In the post-test, 43% of participants changed all 3 responses from their pre-test, 23% changed 2 responses, and 21% changed 1 response; only 13% retained all 3 of the pre-test responses. The results demonstrate the influence of participants’ newly gained knowledge.

After completing all modules, each participant wrote 4 problem statements based on county-specific data. We organized these problem statements into topics and dimensions and counted the frequency with which participants mentioned each topic (Table 3). The 2 most frequently cited topics were overweight/obesity and tobacco use, 2 long-term priorities for TDH interventions. The following are examples of problem statements that demonstrate how participants drew on their data:

**Table 3:** Dimensions of Health Topics Identified in Problem Statements Created During Workshops on Using Secondary Data for Community Health Assessments, Tennessee, 2012

Health Topic	Number of Problem Statements Generated by Workshop Participants	Dimensions
Overweight/obesity	58	Links to chronic disease, poor eating habits
Tobacco use	67	Links to chronic disease, smoking during pregnancy
Cancer	28	By type and sub-population
Heart disease/stroke	27	Hospitalization, sub-populations
Diabetes	27	Prevalence, disparities by sub-population group
Infant mortality	25	Links to low birth weight risk factors
Substance abuse	25	Drugs and alcohol, accidents, hospitalization rates
Oral health/dental care	24	Access, use of emergency department, loss of teeth
Health disparities	16	Multiple causes, race, and sex

- The 2007–2009 mortality rate (per 100,000) for children aged 1 to 4 in [East] County is almost 20% lower than the state rate of 32.6, whereas the 2007–2009 mortality rate (per 100,000) for children aged 15 to 18 in [East] County is almost 30% higher than the state rate of 66.9.
- The 2007–2009 mortality rate (per 100,000) for children aged 5 to 14 in [East] County is 304% higher than the state rate.
- From 2002 to 2006, the rate of emergency department visits for asthma among [Middle] County children (aged 1–17) was 1.5 times greater than the state rate for that group. Charges for inpatient hospitalizations related to asthma for children in the county were nearly twice the average charges for the state.

Counties confirmed the community benefit of the CHA by sharing data and other findings with health, hospital, school, and government leaders in their counties. Ninety-five percent of counties responding to an evaluation administered 9 months after the workshops reported presenting their data to community stakeholders. More than 80% shared data with both county health department staff and community health councils. Half of the counties shared data with hospitals as part of the new CHA requirements for nonprofit hospitals. Counties also indicated that CHA data and problem statements were useful in their selection of a health topic for a new TDH requirement to develop a community-based primary prevention initiative. Participants reported the CHA was most helpful in improving a sense of competency in 3 areas: 1) identifying sources of public health data and information; 2) identifying the health status of populations and the related determinants of health and illness; and 3) adhering to ethical principles in the collection, maintenance, use, and dissemination of data and other information.

## Considerations and Challenges

The CHA process supported counties’ efforts to improve their essential public health services (monitoring health status, diagnosing community health problems, and informing people about health issues), addressed prerequisites to accreditation standards, and improved workforce competencies.

Public health leaders contemplating a CHA that uses the Tennessee program as a template might consider some of the following observations. From a design perspective, imposing limitations on module content was necessary for developing a workshop that could be completed in 2 days. The 2-day time frame required PPA staff to be selective about which data to introduce to participants. Awareness of statewide and region-specific population health disparities, the degree of difficulty in extracting data from websites, and the desire for a statewide focus on TDH priority health issues also influenced data selection. Each module was designed as a completed product, which participants could repeat to further explore data. Workshop leaders clearly stated that the value of secondary data assessment was not to identify causal mechanisms for disease but rather to increase knowledge of the county’s health data and methods for obtaining additional data. A second phase of the CHA, planned for 2012–2013, will introduce community-interactive qualitative assessment methods to clarify causal factors for selected health issues.

Module content and layout was the most time-consuming part of the development process. The program designers had to check methodically the correctness of all secondary data and website sources. Logical clustering of data points and potential responses to trigger questions in each module required careful scrutiny to ensure meaningful findings for participants. Likewise, the staff that created Excel spreadsheets with pre-formatted cells for data entry and automatic calculations of county-to-state or county-to-nation comparisons had to ensure that each cell functioned properly to guarantee a standardized, repeatable process. Designers created a second print page that was automatically populated with inputted and computed data. The modules are now a replicable tool through which counties can monitor population health changes over time for program planning and implementation purposes.

That participants reported being significantly more comfortable with using data by the end of the workshop suggests they will also be more comfortable in using data for decision-making purposes. Multiple counties demonstrated this comfort by creating county fact sheets based on CHA data for communicating population health risks to community members and then engaging community members in planning, developing, and conducting successful population interventions.

We identified 4 categories of implementation challenges: resource limitations, planning and preparation, facility limitations, and communication. Ensuring the availability of such resources as qualified, skilled personnel and computer access was pivotal for success. Developing a CHA approach to meet participants’ time limitations challenged the designers’ ability to balance teaching strategies and learning objectives. Allocating planning time for creating, developing, and testing the modules was also critical. The hands-on learning approach required simplicity in module design to accommodate an audience with varied skills. The choice of the training facility influenced participants’ interaction, an important component of group learning. Computer labs with appropriate software versions and Internet access (without firewalls) and a high level of communication among the many stakeholders were also required to ensure a successful program.

Post-workshop evaluation identified several potential improvements for program development and implementation. The module development stage should use a standard project-management technique to assign time and tasks and allocate sufficient resources for module pre-tests. Designers should consider alternative methods for deciding which modules are developed and used. Workshop participants indicated that time allocated for introduction, module completion, and group debriefing was insufficient. Scheduling flexibility may be required for future workshops; for example, the program planners added a 1-hour pre-workshop session to upgrade participants’ basic computer skills, including skills used for Excel software and navigation of the Internet. A written agreement that specifies follow-up steps to encourage participants to use what they learn is another potential improvement; such an agreement would include a commitment to respond to a post-workshop evaluation, develop county fact sheets, and present data to local audiences.

TDH recognizes CHA as fundamental to the delivery of multiple essential public health services (9). The Tennessee CHA met multiple objectives, including staff training, improvement of internal TDH customer satisfaction, and a process for using secondary data to improve place- and population-based assessment and planning. Given Tennessee’s poor national ranking in multiple health outcomes and health determinants (10), improving public health workforce competence and promoting attention to county-level health indicators will encourage community awareness of local health issues and spur discussions on health improvement. The CHA, seen within the Baldrige Performance Improvement framework, opened new communication between TDH central office sources of data and county health department customers. This communication strengthens local and state strategic planning and creates greater appreciation for data measurement and knowledge management.
